# Germinated Haricot Bean and Teff‐Fortified Barley Porridge for Combatting Pregnant Women's Malnutrition: Optimization Using Mixture Design, Regression Modeling, Contour Plot Analysis, and Nutritional Evaluations

**DOI:** 10.1002/fsn3.70724

**Published:** 2025-08-02

**Authors:** Ramesh Duraisamy, Abera Terefe, Belay Haile Kebede, Betelhem Abera Mengistu

**Affiliations:** ^1^ Department of Chemistry (Industrial Chemistry Division), College of Natural & Computational Sciences Arba Minch University Arba Minch Ethiopia

**Keywords:** barley, haricot bean, mineral, porridge, pregnant women

## Abstract

In Ethiopia, malnutrition among pregnant women, particularly in rural areas, remains a critical public health challenge, necessitating a strong focus on developing innovative food products. This study aimed to develop optimized composite porridge flours for pregnant women by blending germinated haricot beans, teff, and barley, using regression modeling and contour plot analysis. The flours were blended within 30%–60% for barley, 10%–30% for teff, and 30%–60% for germinated haricot beans. The formulated composite flours exhibited proximate composition values (g/100 g) within the ranges of moisture (8.96–9.46), ash (3.46–4.76), crude fat (2.09–3.64), crude fiber (9.16–10.75), crude protein (14.32–17.57), carbohydrate (66.57–69.58), and energy value (355.57–365.92 kcal/100 g). The mineral content (mg/100 g) included Fe (12.12–16.22), Zn (2.07–5.13), Ca (240.88–582.8), Mg (285.98–534.53), and K (1184.85–1599.99). Anti‐nutrient content (mg/100 g) comprised tannins (8.46–13.16) and oxalates (8.77–12.52). The functional properties results (%) encompassed WAC (36.36–56.04) and OAC (35.46–54.65). The sensory evaluations of the porridge showed acceptable scores in appearance, aroma, taste, texture, and overall acceptability. The study revealed that incorporating germinated haricot bean and teff flours into barley flour significantly enhanced its nutritional and functional properties, making it suitable for pregnant women.

## Graphical abstract

In this study, composite flours were developed by blending germinated haricot beans, teff, and barley to create a nutritious porridge for pregnant women in Ethiopia. The formulated flours exhibited favorable proximate composition, mineral content, and functional properties, enhancing their suitability as supplementary food for combating malnutrition among pregnant women.

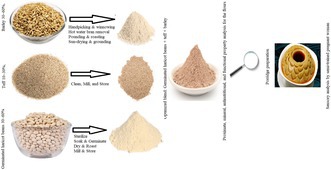

## 1 Introduction

Malnutrition remains a critical public health issue in Africa, contributing to the deaths of 3.5 million women of reproductive age annually. This problem disproportionately affects women in low‐ and middle‐income households, particularly pregnant women, and adversely impacts childbirth outcomes (Jiang et al. 2022). In Ethiopia, poor nutrition has become a significant health challenge, with 71.4% of the population dependent on cereal‐based diets deficient in essential nutrients, while less than 2.7% consume animal‐based foods (Boateng et al. 2025; Bajo et al. 2021). This reveals the urgent need to improve dietary diversity and nutrient intake, especially among vulnerable groups.

Barley, a traditional Ethiopian crop, offers a sustainable solution as a rich source of carbohydrates, β‐glucans (soluble fiber), and essential minerals like selenium. When combined with nutrient‐dense legumes, it forms a complete nutritional base. Sprouting haricot beans significantly enhances their bioavailability, reducing anti‐nutrients (e.g., phytates and tannins) by up to 50% while increasing protein digestibility (20%–30%), iron (40%), and zinc (25%) absorption (Wodajo and Emire 2022; Dhanya et al. 2024). This processing method is particularly valuable for pregnant women, who require 50% more iron and 40% more protein daily to support fetal development and prevent anemia, as per the WHO/FAO Recommended Dietary Allowances (RDA) for pregnant women (Fontanini et al. 2025; Alemayehu et al. 2021).

The composite flour is nutritionally optimized for pregnancy and formulated to meet WHO/FAO and Ethiopian National Nutrition Guidelines (2020) for maternal nutrition. It provides 14–18 g/100 g protein (fulfilling ~30%–40% of increased pregnancy RDA), 12–16 mg/100 g iron (meeting 60%–80% of enhanced needs), and folate from teff (400 μg DFE/day) for neural tube development. Barley contributes low‐glycemic carbohydrates to sustain energy and reduce gestational diabetes risk, creating a balanced solution for maternal and fetal health (Ogunniran et al. 2024; Punzalan et al. 2025).

In Ethiopia, traditional diets rely heavily on cereals and legumes, yet rural populations often face limited access to processed foods and animal protein due to cost barriers. Incorporating protein‐ and mineral‐rich foods like beans and teff could help bridge nutritional gaps (Singh et al. 2015). While cereals provide carbohydrates and energy, excessive reliance on them leads to deficiencies, particularly among pregnant women who require higher‐quality diets. Additionally, incorporating cereals and beans into one's diet has been shown to provide a rich source of macro‐ and micronutrients (Habineza et al. 2025).

Previous research indicates that 41.2%–46.3% of pregnant women in rural southern Ethiopia suffer undernutrition, with the Gofa Zone Health Department reporting prevalence rates of 41.6% in Geze Gofa town (Hussain and Sharma 2025). This is attributed mainly to monotonous, cereal‐heavy diets. Haricot beans and teff grains, rich in proteins and minerals, offer a viable solution for improving dietary quality. However, a lack of awareness about nutritional values hinders their optimal use (Alemayehu et al. 2021; Mmbando and Missanga 2024). To address this gap, this study aims to develop nutrient‐dense composite flour by blending sprouted haricot beans, teff, and barley using a mixture design. The formulation aligns with traditional porridge preparation methods in Geze Gofa town, ensuring cultural acceptability.

## 2 Materials and Methods

### 2.1 Raw Material Collection

White haricot bean grains ( *Phaseolus vulgaris* L.), two‐row hulled barley ( *Hordeum vulgare* L.), and red teff ( *Eragrostis tef* ) grains were purchased from Bulki Market in Gofa Zone, Ethiopia, and transported to Arba Minch University's Food Laboratory, Arba Minch, Ethiopia. The ingredients were selected based on their exceptional nutritional profiles, local availability, and cultural acceptability.

### 2.2 Raw Material Preparation

#### 2.2.1 Preparation of Barley Flour

Barley grains (1 kg) were cleaned to remove the defective grains, stones, and other debris by handpicking, and then it was winnowed to separate from dust. After cleaning, it was soaked in hot water for about 5 min to remove the bran from the grain. The moistened grain was dehulled to remove the bran from the grain by pounding the grains in a mortar with a pestle. Then, the grains were dried in sunlight for 2 days to separate the husks from the dried grains; it was roasted carefully in a large traditional stove with firewood on low heat, dehulled again slightly, and winnowed to remove the remaining husks (Thornburg and Valent 2024).

The grains were ground using a community milling machine (a small amount of the studied sample was ground in the mill) before grinding the sample to avoid contamination, which was more common in the study area. After the grains were milled, the flour was sieved to separate coarse solid pieces from it by using a sieve with a sieving size of 0.5 mm. Finally, the resulting flours were stored in polyethylene plastic bags, and then nutrients, antioxidants, and functional properties were analyzed (Hussain and Biswas 2024).

#### 2.2.2 Preparation of White Haricot Bean

The germination process for white haricot beans (Awash‐1 variety) was conducted under controlled conditions to ensure reproducibility and food safety. A sample of beans (1 kg) was carefully hand‐sorted to remove defective grains and debris, then surface‐sterilized by soaking in 1% (v/v) food‐grade hydrogen peroxide solution for 10 min to minimize microbial contamination. The sterilized beans were rinsed thoroughly and soaked in sterile distilled water (1:3 w/v ratio) at 25°C for 12 h to initiate sprouting. After draining, the beans were spread evenly on sterile perforated trays lined with moistened filter paper and germinated at 25°C with 85% relative humidity (monitored using a hygrometer) in a dark, ventilated cabinet for 48 h (Cimini et al. 2024).

The beans were rinsed with sterile distilled water every 12 h to prevent mold growth. Germination was halted by oven‐drying at 50°C for 24 h until the moisture content reached ≤ 10% (verified by the AOAC 925.10 gravimetric method), after which the hulls were manually removed. The dried beans were then roasted at 120°C for 5 min in a traditional stove to inactivate residual enzymes, milled using a community mill sterilized with 70% ethanol, and sieved through a 0.5 mm mesh to obtain uniform flour. Finally, the flour was stored in vacuum‐sealed polyethylene bags at 4°C until analysis (Ogunniran et al. 2024).

#### 2.2.3 Preparation of Teff Flour

Red teff grains (1 kg) were used in the current study because they were more available in the study area. Also, an earlier study by Dhanya et al. (2024) indicated that mixed teff has more micro‐ and macronutrient contents. The teff grains were manually cleaned by winnowing and removing trash, dust, and other impurities. The cleaned grain was ground to flour using the community's milling machine and stored in polyethylene plastic bags until further processing and analysis.

### 2.3 Experimental Design and Data Analysis of Composite Flour Formulation

The study employed a 9‐run extreme vertices mixture design generated by Minitab software (v. 19.1, USA) to evaluate flour formulations systematically. The design incorporated compositional constraints of 30%–60% barley, 10%–30% teff, and 30%–60% germinated haricot bean, with 100% barley flour serving as the control (Table 1). These boundaries were established based on nutritional composition data from literature (Abera et al. 2024, 2025) with modifications to ensure nutritional adequacy and cultural appropriateness. The measured response variables included proximate composition (protein, fat, fiber, ash, carbohydrates, and moisture content), mineral profile (iron, zinc, calcium, and potassium), functional properties (water and oil absorption capacities), and antinutrient levels (tannins and oxalates). The D‐optimal mixture design approach was specifically selected to identify the optimal flour blend that would simultaneously: (1) maximize protein content, essential mineral concentrations, and functional properties; (2) minimize antinutrient content; while (3) maintaining carbohydrate and moisture levels.

**TABLE 1:** Proximate composition (% w/w) and energy values (kcal/100 g) of raw materials and composite flours.

Proximate composition							
	Moisture	Ash	Fat	Fiber	Protein	CHO	Energy
RHB	12.37 ± 0.35^a^	3.26 ± 0.17^c^	3.54 ± 0.12^b^	6.34 ± 0.01^c^	22.05 ± 0.45^b^	60.31 ± 0.00^c^	359.89 ± 0.03^b^
GHB	9.28 ± 0.01^d^	5.22 ± 0.10^a^	2.66 ± 0.20^d^	7.11 ± 0.02^d^	24.94 ± 0.18^a^	57.54 ± 0.18^d^	355.31 ± 0.01^d^
Teff	11.02 ± 0.01^c^	3.49 ± 0.01^b^	3.54 ± 0.01^c^	12.89 ± 0.21^a^	11.13 ± 0.14^c^	69.89 ± 0.14^b^	364.20 ± 0.03^a^
Barley (C_0_)	12.03 ± 0.39^b^	2.46 ± 0.02^d^	4.45 ± 0.03^a^	9.37 ± 0.02^b^	9.72 ± 0.02^d^	72.26 ± 0.35^a^	359.79 ± 1.38^c^

Flour mixture ratio										
Code	A	B	C							
S_1_	30	10	60	8.96 ± 0.01^f^	4.76 ± 0.06^a^	2.09 ± 0.01^h^	9.56 ± 0.03^e^	17.57 ± 0.05^a^	66.62 ± 0.01^g^	355.57 ± 0.02^i^
S_2_	35	15	50	9.12 ± 0.21^e^	4.43 ± 0.02^c^	2.99 ± 0.01^d^	9.29 ± 0.01^g^	16.26 ± 0.02^b^	67.20 ± 0.02^f^	360.75 ± 0.03^e^
S_3_	40	30	30	9.66 ± 0.01^a^	3.77 ± 0.11^e^	3.64 ± 0.02^a^	10.25 ± 0.01^b^	15.34 ± 0.01^g^	67.95 ± 0.01^d^	365.92 ± 0.05^a^
S_4_	40	20	40	9.45 ± 0.02^c^	4.16 ± 0.08^d^	2.58 ± 0.00^e^	9.77 ± 0.01^c^	15.86 ± 0.01^d^	67.95 ± 0.03^d^	358.46 ± 0.04^g^
S_5_	50	15	35	9.51 ± 0.01^b^	3.49 ± 0.02^g^	2.14 ± 0.06^g^	9.55 ± 0.01^e^	15.43 ± 0.01^f^	69.43 ± 0.03^b^	358.70 ± 0.28^f^
S_6_	30	30	40	9.35 ± 0.01^d^	4.55 ± 0.05^b^	3.57 ± 0.01^b^	10.75 ± 0.05^a^	15.96 ± 0.02^c^	66.57 ± 0.01^h^	358.25 ± 0.11^h^
S_7_	35	25	40	9.42 ± 0.03^c^	3.68 ± 0.01^f^	3.53 ± 0.01^b^	9.65 ± 0.01^d^	15.52 ± 0.01^e^	67.85 ± 0.04^e^	365.25 ± 0.04^b^
S_8_	60	10	30	9.52 ± 0.01^b^	3.41 ± 0.22^i^	3.11 ± 0.03^c^	9.16 ± 0.03^h^	14.32 ± 0.01^i^	69.28 ± 0.02^c^	362.39 ± 0.36^c^
S_9_	40	25	35	9.46 ± 0.03^c^	3.46 ± 0.03^h^	2.54 ± 0.00^f^	9.46 ± 0.01^f^	14.96 ± 0.01^h^	69.58 ± 0.02^a^	361.02 ± 0.90^d^
C.V. %				0.36	1.96	8.56	1.50	1.06	0.37	0.38

*Note:* Results expressed in mean ± SD; Mean does not share the same superscripts in the column are significantly different (*p* < 0.05).

Abbreviations: A, amount of barley flour (g); B, the amount of teff flour (g); C, the amount of germinated haricot bean flour (g); C.V %, coefficient of variation (%); C_0_, control sample (100% barley); CHO, carbohydrate; GHB, germinated haricot bean flour; RHB, raw haricot bean flour; S_1_–S_9_, sample one to nine.

### 2.4 Preparation of Porridge

The porridge samples were prepared following traditional methods under controlled cooking conditions. In a standardized 2 L stainless steel cooking pot, 750 mL of potable water (equivalent to three metric cups at 250 mL/cup) was heated to a boil (100°C at sea level) on a gas burner set to medium heat (180°C surface temperature). Once boiling, 5 g of iodized table salt (1 level teaspoon) was added and dissolved completely. Then, 250 g of composite flour (equivalent to two metric cups at 125 g/cup, sieved through a 0.5 mm mesh) was gradually added in 50 g increments at 30‐s intervals while stirring constantly with a stainless steel ladle at 60 rpm to prevent lump formation (Thornburg and Valent 2024).

The mixture was maintained at 95°C for 12 min with continuous stirring until achieving a target viscosity of 800 cP (measured by Brookfield viscometer at 60°C). To prevent sticking, 50 mL of room‐temperature (25°C) water was incorporated while stirring vigorously for 30 s, followed by additional cooking for precisely 5 min at the same temperature until complete water absorption. For seasoning, 60 mL of refined sunflower oil (1/4 metric cup) was uniformly drizzled over the hot porridge while stirring, immediately followed by 2 g of standardized chili powder (1 level teaspoon) distributed in three equal portions at 1‐min intervals. The final product was immediately transferred to presterilized, food‐grade polyethylene containers (500 mL capacity) and covered with sterile lids to retain heat and moisture (Hussain and Sharma 2025; Jeong et al. 2025). Both the optimized composite flour porridge and control samples (prepared simultaneously under identical conditions) were served to panelists at 65°C within 15 min (Ogunniran et al. 2024).

### 2.5 Determination of Proximate Compositions in Raw Materials and Composite Flours

This research employed standardized analytical methods to determine the proximate composition of the composite flour samples. Moisture content was quantified using the AOAC 925:10 methods, where samples were oven‐dried and weight loss measured. Ash content was determined through incineration at 550°C following AOAC 923:03 guidelines. Crude fat extraction utilized the Soxhlet apparatus with petroleum ether as per AOAC 945:16, while crude fiber analysis involved acid/alkali digestion and subsequent incineration at 600°C. Protein content was measured via the Kjeldahl method, employing acid digestion and titration with a 6.25 conversion factor. Total carbohydrates were calculated by difference, subtracting the sum of moisture, ash, protein, and fat percentages from 100%. Energy values were derived using Atwater factors (4 kcal/g for protein and carbohydrates, 9 kcal/g for fat). All analyses were conducted in triplicate following established AOAC (2000) protocols, with results expressed as percentages or kcal per 100 g sample.

### 2.6 Determination of Mineral Content

For mineral analysis, samples were ashed according to AOAC (2000) guidelines, and then digested using a nitric‐perchloric acid mixture until clear solutions were obtained. The digested samples were filtered, diluted to volume, and analyzed by atomic absorption spectroscopy (BUCK 240 VGA) at element‐specific wavelengths, with concentrations calculated using calibration curves. Tannin content was determined through the vanillin‐HCl method, where samples were extracted with acidified methanol, centrifuged, and reacted with vanillin reagent before spectrophotometric measurement at 500 nm. Oxalate analysis followed AOAC protocols involving sulfuric acid extraction and titration with potassium permanganate. All measurements were performed in triplicate, with results calculated using established formulas that accounted for blank corrections and dilution factors. The mineral, tannins, and oxalate content were expressed as mg/100 g sample.

### 2.7 Functional Properties of Flour Samples

The study evaluated the functional properties of flour samples through water and oil absorption capacity analyses. For water absorption capacity (WAC), 1 g samples were mixed with distilled water, allowed to hydrate for 30 min, then centrifuged at 4000 rpm for 15 min; the absorbed water percentage was calculated from weight differences before and after centrifugation Equation (1).

(1) $$\mathrm{WAC}\;\left(\%\right)=\left(W_{2}-W_{1}\right)/\left(W_{0}\right)\times 100$$

W_1_ is the weight of the sample before centrifugation (g), W_2_ is the weight of the sample after centrifugation (g), and W_0_ is the weight of the original sample taken (g).

Similarly, oil absorption capacity (OAC) was determined by mixing samples with vegetable oil, centrifuging at 4000 rpm for 25 min, and quantifying retained oil in Equation (2). Both methods, adapted from established protocols of Fontanini et al. (2025) and Ogunniran et al. (2024) employed precise gravimetric measurements to assess hydration and lipid retention properties critical for product formulation. Results were expressed as percentage absorption relative to original sample weights (W_0_).

(2) $$\mathrm{OAC}\;\left(\%\right)=\left(W_{2}-W_{1}\right)/\left(W_{0}\right)\times 100$$

### 2.8 Evaluation of Sensory Properties of Porridge Samples

Four optimized barley porridge formulations along with a control sample were subjected to sensory evaluation using a 5‐point hedonic scale (1 = dislike extremely, 5 = like extremely) to assess appearance, aroma, taste, texture, and overall acceptability. The study employed 50 semi‐trained pregnant women panelists recruited from Bulki Health Center, randomly assigned to evaluate the samples presented in identical containers labeled with three‐digit random codes to prevent bias. Researchers and health workers assisted participants, particularly those with limited literacy, to ensure proper evaluation while maintaining standardized testing conditions. Between sample tastings, panelists cleansed their palates with potable water to minimize carryover effects. Participants ranked all five porridge samples based on their preference scores, which were subsequently collected for statistical analysis of sensory acceptability (Cimini et al. 2024; Molapisi et al. 2024).

### 2.9 Statistical Analysis

The data were subjected to analysis of variance (ANOVA) with a predetermined significance threshold of *α* = 0.05, and Tukey's least significant difference test using Minitab software. The study utilized mixture regression to fit the special cubic model, considering *p*‐values below 0.05 as statistically significant. Positive coefficients were used to identify synergistic interaction terms, pinpointing the “sweet spot” for optimal responses. Regression analysis was conducted using Minitab software to evaluate model significance, lack of fit, R‐squared (*R* ^2^), and adjusted R‐squared (adjusted‐*R* ^2^) to assess the fitness of the model (Alemayehu et al. 2021; Abera et al. 2024).

## 3 Results and Discussion

### 3.1 Proximate Compositions of the Raw Materials

The proximate composition analysis of the flour samples (Table 1) revealed significant nutritional variations essential for formulating optimized composite flour for pregnant women. HB (12.37% ± 0.35%) and barley (12.03% ± 0.39%) exhibited the highest moisture content, which may influence shelf stability, while GHB (9.28% ± 0.01%) demonstrated superior storage potential due to its lower moisture level. These findings align with Kemal et al. (2025), who attributed reduced moisture in germinated flours to water utilization during processing. The ash content was highest in GHB (5.22% ± 0.10%), indicating rich iron, calcium, and zinc reserves—critical micronutrients for fetal development and maternal health—while barley (2.46% ± 0.02%) showed the lowest mineral content.

Fat content analysis has shown barley (4.45% ± 0.03%) and HB (3.54% ± 0.12%) as valuable sources of essential fatty acids, whereas GHB has lower fat content (2.66% ± 0.20%). The current study found teff's crude fiber content (12.89% ± 0.21%) to be significantly higher than literature values (3%–4%) by Belarbi et al. (2025), likely due to varietal differences, analytical methods, or milling processes. Protein levels were significantly higher in GHB (24.94% ± 0.18%) and HB (22.05% ± 0.45%), meeting the elevated protein demands of pregnancy (71 g/day), while teff (11.13% ± 0.14%) and barley (9.72% ± 0.02%) served as secondary sources.

Carbohydrate analysis of barley (72.26% ± 0.35%) and teff (69.89% ± 0.14%) as primary energy providers, though their high glycemic potential necessitates careful inclusion for women at risk of gestational diabetes. In contrast, HB (60.31% ± 0.00%) and GHB (57.54% ± 0.18%) offered lower‐carb alternatives. The current results align with Derese and Shimelis (2022) and Feyissa et al. (2022) on barley‐based composites.

### 3.2 Proximate Composition of the Composite Food Formulation

The composite food formulation was optimized using a mixture design approach, where polynomial regression models Equation (3) quantified how nutritional properties responded to varying ingredient ratios.

(3) $$Y=\sum_{i=1}^{n}\text{βixi}+\sum_{i=1}^{n-1}\;\sum_{j=i+1}^{n}\text{βijxixj}+\epsilon$$

where: *Y*: Response variable, *X*_*i*_ and *X*_*j*_: proportions of raw materials, *βi*: Linear coefficients, *βij*: Interaction coefficients, *n*: variables, and *ϵ*: Random error term.

#### 3.2.1 Moisture

The moisture characteristics of the composite flour formulations were thoroughly analyzed using polynomial regression modeling Equation (4). The regression equation revealed distinct moisture behavior patterns among the components, with barley flour showing the most substantial positive influence on moisture content due to its high native water retention capacity. In contrast, germinated haricot bean flour demonstrated a significant drying effect, which can be attributed to the moisture reduction during germination.

(4) $$\begin{array}{cc} \text{Moisture}= & +0.1613\;A+0.8553\;B-0.1467\;C-0.0189\;\mathrm{AB}\\ & -0.0022\;\mathrm{AC}-0.0194\;\mathrm{BC}-0.0004\;\mathrm{ABC} \end{array}$$

**FIGURE 1:**
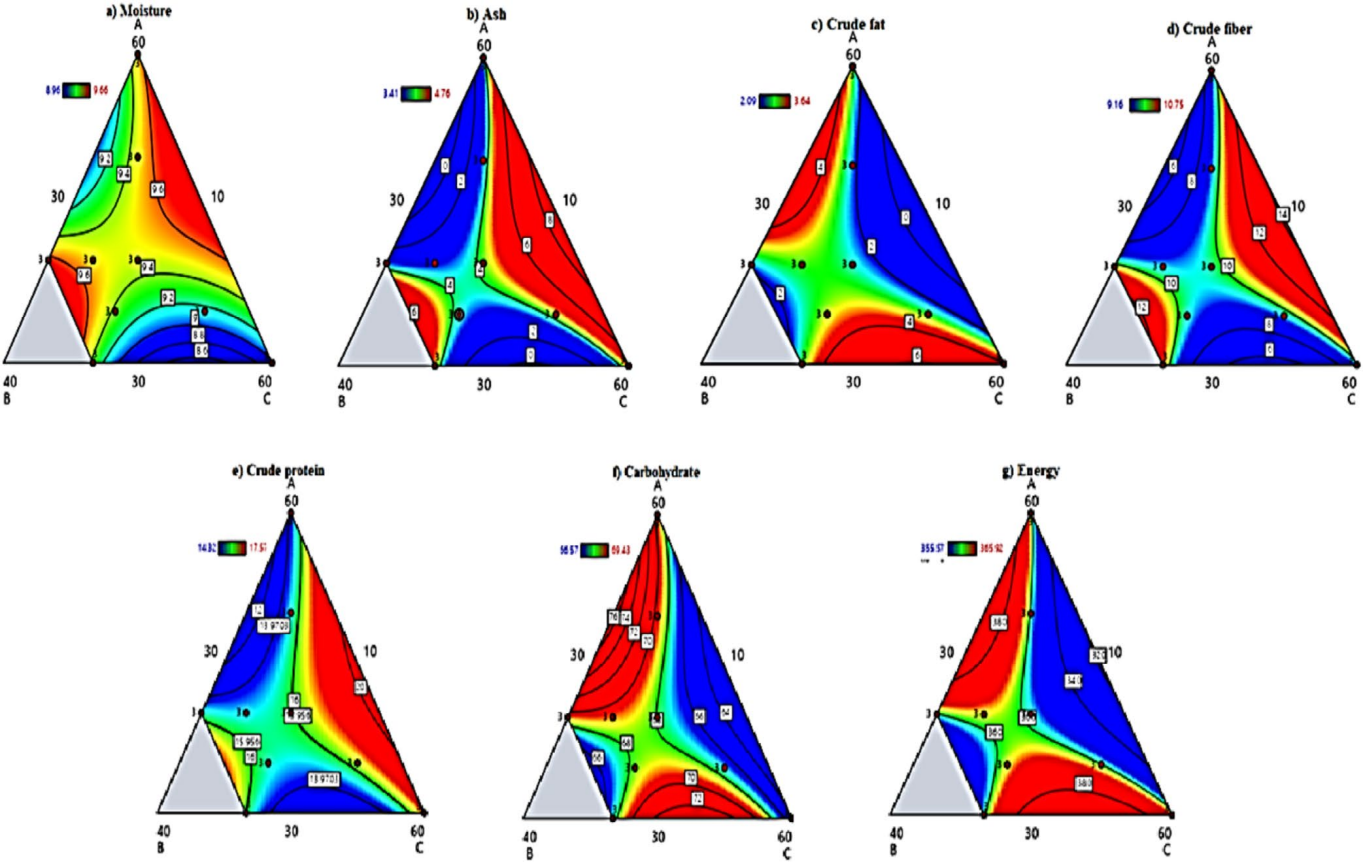
Contour plots of proximate components of barley‐teff‐GHB composite flours.

The mathematical model also identified significant interaction effects, particularly the synergistic moisture reduction when germinated haricot bean is combined with barley flour. An ideal blend composition was determined through optimization analysis, consisting of 51.8% germinated haricot bean, 30.6% barley, and 17.6% teff flours, which achieved a remarkably low moisture content of 8.6% (Figure 1). This substantial moisture reduction stems from three key mechanisms: the structural changes in haricot beans during germination that reduce their water retention capacity, the disruption of starch‐water networks in the cereal flours by components from the germinated beans, and competitive water binding between the different flour fractions. The achieved moisture level below 12% provides significant technological advantages, including extended shelf‐life through inhibition of microbial growth, improved processing characteristics due to enhanced powder flowability, and greater storage stability with reduced risk of caking.

#### 3.2.2 Ash

The polynomial regression analysis, Equation (5) revealed significant differences in mineral contributions among flour components, with germinated haricot bean (GHB) demonstrating the strongest positive effect on ash content (coefficient = +5.9637), followed by teff (+0.4237). The three‐factor interaction term (ABC = +0.0031) indicated synergistic mineral retention in ternary blends, while binary combinations showed varied effects: barley‐teff (AB = −0.1466) and barley‐GHB (BC = −0.1428) exhibited antagonism, whereas teff‐GHB (AC = −0.0107) had minimal interaction. These results explain why optimized composite blends achieved 5.03 g/100 g ash content—substantially higher than control samples (100% barley, 2.46 g/100 g) (Figure 1) and previous wheat‐potato‐GHB formulations (3.33%, Abera et al. 2025). The superior mineral content of GHB‐containing blends can be attributed to germination‐induced phytate hydrolysis, which increases mineral bioavailability (Hussain and Sharma 2025; Kemal et al. 2025).

(5) $$\begin{array}{cc} \mathrm{Ash}= & +0.4237\;A+5.9637\;B+0.4203\;C-00.1466\;\mathrm{AB}\\ & -0.0107\;\mathrm{AC}-0.1428\;\mathrm{BC}+0.0031\;\mathrm{ABC} \end{array}$$

Notably, while optimizing moisture characteristics, the composite flours maintained an ash content range of 2.5–4.9 g/100 g, indicating good mineral retention. This suggests that the germination process enhances mineral bioavailability without compromising content, and that the ternary blending approach successfully balances moisture control with nutritional quality.

#### 3.2.3 Crude Fat

The regression analysis, Equation (6), demonstrated that all flour components significantly influenced the crude fat content of the composite formulations, which ranged from 2.09 ± 0.01 g/100 g to 3.64 ± 0.01 g/100 g. The polynomial equation revealed that barley flour had the strongest positive effect on fat content (+2.4221 coefficient), followed by teff flour (+0.2657). At the same time, germinated haricot bean (GHB) showed a negative effect (−0.2912) due to its inherently lower fat content. This pattern was reflected in the experimental results, where increasing proportions of barley and teff led to higher fat levels, while GHB incorporation reduced overall fat content.

(6) $$\begin{array}{cc} \text{Crude}\;\mathrm{fat}= & +0.2657\;A+2.4221\;B-0.2912\;C+0.0482\;\mathrm{AB}\\ & -0.0149\;\mathrm{AC}-0.0491\;\mathrm{BC}-0.0008\;\mathrm{ABC} \end{array}$$

The fat content of barley‐teff blends enhances palatability through improved mouthfeel and flavor release. In contrast, the optimized tertiary blend (44.3% barley, 25.3% teff, 30.4% GHB) achieves a moderate fat level (4.15 g/100 g) (Figure 1), balancing sensory quality with WHO cardiovascular guidelines. Barley flour's higher native fat content (4.45% vs. teff's 3.54% and GHB's 2.66%) was offset by negative interaction terms in blends (−0.1466 AB, −0.1428 bc). In contrast, the positive ABC term (+0.0031) indicates that balanced inclusion of all three components optimizes fat and mineral retention. Germinated haricot bean (GHB) proved critical for lower‐fat, mineral‐enriched formulations, particularly maternal nutrition (Habineza et al. 2025).

#### 3.2.4 Crude Fiber

The fiber content analysis of the composite flour formulations revealed important trends and interactions between ingredients. The fiber levels ranged from 9.16 ± 0.03 g/100 g to 10.75 ± 0.05 g/100 g across different blends, consistently exceeding the control sample's fiber content. This enhancement is primarily attributed to high‐fiber teff flour (12.89 ± 0.21 g/100 g crude fiber) in the formulations. Formulation S6 (30% teff, 30% barley, 40% GHB) demonstrated the highest crude fiber content among the tested combinations. S_8_ (60% teff, 10% barley, 30% GHB) showed the lowest, indicating an antagonistic effect between barley and teff components at specific ratios. The relationship between flour components and fiber content was mathematically modeled through polynomial regression Equation (7), which revealed several key insights. Barley flour emerged as the dominant contributor to fiber content (coefficient + 5.4976), likely due to its rich β‐glucan content (3.2–4.1 g/100 g).

(7) $$\begin{array}{cc} \text{Crude fiber}= & -0.3246\;A+5.4976\;B+0.3194\;C-0.1266\;\mathrm{AB}\\ & -0.0042\;\mathrm{AC}-0.1253\;\mathrm{BC}+0.0026\;\mathrm{ABC} \end{array}$$

However, the negative coefficient for the barley‐teff interaction term (−0.1266 AB) suggests that excessive combinations of these two flours may reduce overall fiber retention, possibly due to competing water‐binding properties. Notably, the positive three‐factor interaction term (+0.0026 ABC) indicates that carefully balanced ternary blends (such as 44.92% barley, 10.24% teff, and 44.84% GHB) can achieve optimal fiber content up to 12.04 g/100 g, exceeding WHO's recommended daily fiber intake levels. The current study also identified important moisture‐fiber trade‐offs, with GHB's moisture‐reducing effect (9.28%) potentially conflicting with the water‐binding properties of barley (12.03%) and teff (11.02%).

#### 3.2.5 Crude Protein

A polynomial regression model Equation (8) effectively captured these protein dynamics, revealing GHB as the dominant protein source (coefficient + 5.0999) and identifying optimal protein complementation in 40%–50% barley‐GHB blends that achieved balanced amino acid profiles. The germination process significantly enhanced the protein quality of haricot beans, increasing content from 22.05 ± 0.45 g/100 g to 24.94 ± 0.18 g/100 g after 48 h, likely due to proteolytic activation and accumulation of nonprotein nitrogen compounds. This protein enrichment was reflected in the composite formulations, where protein levels varied systematically based on component ratios. Formulation S_1_, containing 60% GHB, showed the highest protein content (17.36 ± 0.05 g/100 g), while S_8_, with only 30% GHB, had the lowest (14.50 ± 0.01 g/100 g).

(8) $$\begin{array}{cc} \text{Crude protein}= & -0.2931\;A+5.0999\;B+0.4203\;C-0.1157\;\mathrm{AB}\\ & +0.0029\;\mathrm{AC}-0.1182\;\mathrm{BC}+0.0024\;\mathrm{ABC} \end{array}$$

While GHB‐barley combinations showed mild antagonism, the overall ternary interaction indicated positive synergism in balanced formulations. Nutritionally, the 51.8% GHB composite provided 17.57 ± 0.05 g/100 g protein, fulfilling over 90% of WHO/UNICEF pregnancy requirements. The germination process enhanced protein quality and reduced carbohydrate content through the enzymatic breakdown of complex carbohydrates. Although barley's phytate content somewhat limits mineral bioavailability, the blends maintained superior iron levels compared to other composite formulations. These findings position GHB‐based flours as valuable for addressing maternal malnutrition, providing complete protein and bioavailable iron during critical pregnancy stages while offering metabolic benefits through reduced carbohydrate content.

#### 3.2.6 Carbohydrates

The present study revealed significant variations in carbohydrate content across the composite flour formulations, ranging from 66.57 ± 0.01 g/100 g to 69.43 ± 0.03 g/100 g, with statistically significant differences observed (*p* < 0.05). These values were consistently lower than the control sample containing 100% barley flour (72.26 ± 0.35 g/100 g), confirming that cereal components—particularly barley—served as the primary carbohydrate source in the blends. The carbohydrate dynamics were effectively modeled through polynomial regression analysis Equation (9), which showed that higher proportions of barley increased carbohydrate content. At the same time, greater amounts of teff and germinated haricot bean (GHB) decreased carbohydrate levels.

(9) $$\begin{array}{cc} \text{Carbohydrates}= & +0.1441\;A-8.3746\;B-0.2786\;C+0.2308\;\mathrm{AB}\\ & +0.0309\;\mathrm{AC}+0.2291\;\mathrm{BC}-0.0052\;\mathrm{ABC} \end{array}$$

The regression model revealed several key interaction effects: while teff and GHB individually showed negative coefficients (−0.2786 and −0.2786, respectively), indicating their carbohydrate‐reducing properties, their combinations with barley (AB and BC terms) demonstrated synergistic effects that partially offset this reduction. Notably, the three‐factor interaction (ABC term) exhibited an antagonistic effect, resulting in lower carbohydrate levels than predicted by simple additive models. These findings align with previous research reporting carbohydrate contents of 53.17–71.74 g/100 g in similar cereal‐legume composites and meet WHO recommendations for pregnancy (55 g/day in the second trimester; 65 g/day in the third trimester). The reduced carbohydrate content in GHB‐containing formulations can be attributed to the enzymatic hydrolysis of starches during germination, which breaks down complex carbohydrates into simpler, more digestible forms. This characteristic, combined with barley's β‐glucans and teff's resistant starch content, suggests these composite flours may offer favorable glycemic properties for maternal nutrition, particularly in contexts requiring careful blood sugar management.

#### 3.2.7 Energy

The present study investigated the energy profiles of composite flour formulations containing germinated haricot bean (GHB), teff, and barley, revealing important nutritional characteristics. Individual component analysis showed teff flour possessed the highest energy density (364.20 ± 0.03 kcal/100 g), followed by barley (359.79 ± 1.38 kcal/100 g) and GHB (355.31 ± 0.01 kcal/100 g), with these values strongly correlating with their respective carbohydrate contents. The energy dynamics of composite blends were mathematically modeled through polynomial regression, revealing complex interaction patterns Equation (10).

(10) $$\begin{array}{cc} \text{Energy}= & +2.9874\;A-35.0777\;B+3.1875\;C+0.8943\;\mathrm{AB}\\ & -0.0230\;\mathrm{AC}+0.8854\;\mathrm{BC}-0.0179\;\mathrm{ABC} \end{array}$$

While barley flour exhibited the strongest individual influence on energy content, binary combinations of barley‐teff and barley‐GHB demonstrated synergistic positive effects, enhancing overall caloric density. Interestingly, three‐component blends showed a slight energy reduction, suggesting that complete formulations optimize other nutritional parameters at a marginal energy cost. All tested formulations exceeded pregnancy‐specific energy recommendations, providing 355–364 kcal/100 g to meet 78%–80% of additional gestational energy requirements through typical consumption patterns.

### 3.3 Regression Mixture Analysis for Proximate Composition of Composite Flour

The current study employed comprehensive statistical analyses to evaluate the nutritional composition of composite flour formulations. The models demonstrated excellent fit for most parameters, as evidenced by coefficients of determination (*R* ^2^) and low coefficients of variation (C.V. < 10%), indicating both strong predictive power and high experimental reliability. Statistical significance was confirmed through large *F*‐values and small *p*‐values (< 0.05) across most analyses (Table 2).

**TABLE 2:** ANOVA parameters for the proximate composition of composite flour samples.

Source	Moisture	Ash	Fat	Fiber	Protein	CHO	Energy
Regression	< 0.001	< 0.001	< 0.001	< 0.001	< 0.001	< 0.001	0.002
Linear	< 0.001	< 0.001	0.075	< 0.001	< 0.001	0.005	0.023
Quadratic	0.001	< 0.001	< 0.001	< 0.001	0.001	0.001	< 0.001
Special Cubic	0.005	< 0.001	0.004	< 0.001	0.002	0.006	0.042
Lack‐of‐fit	0.127	0.254	0.089	0.073	0.211	0.055	0.332
*R* ^2^	0.980	0.981	0.845	0.929	0.972	0.978	0.892
Adj. *R* ^2^	0.964	0.975	0.798	0.907	0.964	0.843	0.830

Abbreviations: CHO, carbohydrates; *R* ^2^, coefficient of determination.

### 3.4 Mineral Analysis of Raw Materials and Composite Flours

Minerals are vital in supporting normal physiological functions, growth, and development. The current study evaluated that GHB and teff contained significantly higher mineral concentrations than barley. Despite barley's lower micronutrient density, its 30%–60% incorporation in composite blends offered distinct functional and metabolic advantages. Specifically, formulations containing 40%–50% barley demonstrated optimal water absorption (52.3% ± 1.8%), making them particularly suitable for porridge preparation compared to teff‐only blends (38.2% ± 1.2%) (Table 3).

**TABLE 3:** Mineral compositions (mg/100 g) of raw materials and composite flours.

Mineral composition					
Samples	Fe	Zn	Ca	Mg	K
RHB	10.37 ± 0.45^c^	7.31 ± 0.06^b^	588.00 ± 2.79^b^	343.70 ± 4.53^c^	1419.81 ± 0.94^c^
GHB	14.26 ± 0.00^b^	8.86 ± 0.06^a^	672.76 ± 1.15^a^	567.20 ± 1.18^a^	1797.80 ± 0.54^a^
Barley (C_o_)	9.08 ± 0.25^d^	3.24 ± 0.14^d^	73.89 ± 0.25^d^	305.52 ± 0.16^d^	1201.55 ± 0.54^d^
Teff	17.21 ± 0.00^a^	5.63 ± 0.02^c^	171.23 ± 0.21^c^	424.20 ± 1.15^b^	1458.81 ± 0.14^b^

Flour mixture ratio								
Code	A	B	C					
S_1_	30	10	60	16 ± 0.01^b^	5.07 ± 0.02^b^	582.8 ± 0.19^a^	534.53 ± 0.21^a^	1599.99 ± 1.00^a^
S_2_	35	15	50	15.43 ± 0.01^d^	3.27 ± 0.01^e^	491.78 ± 0.59^b^	470.56 ± 0.01^b^	1561.97 ± 0.01^b^
S_3_	40	30	30	14.92 ± 0.01^e^	5.13 ± 0.01^a^	332.51 ± 0.01^g^	361.50 ± 0.06^g^	1451.20 ± 0.54^e^
S_4_	40	20	40	14.83 ± 0.01^g^	4.12 ± 0.02^c^	400.69 ± 0.05^e^	406.59 ± 0.01^e^	1454.28 ± 1.00^d^
S_5_	50	15	35	13.47 ± 0.02^h^	2.25 ± 0.02^g^	320.78 ± 0.57^h^	346.29 ± 0.44^h^	1386.65 ± 0.00^h^
S_6_	30	30	40	16.22 ± 0.01^a^	4.06 ± 0.01^d^	446.50 ± 0.00^c^	444.35 ± 0.53^c^	1503.58 ± 1.00^c^
S_7_	35	25	40	15.52 ± 0.01^c^	2.97 ± 0.01^f^	423.60 ± 0.58^d^	425.47 ± 0.01^d^	1443.48 ± 0.04^f^
S_8_	60	10	30	12.12 ± 0.01^i^	2.07 ± 0.02^h^	240.88 ± 0.55^i^	285.98 ± 0.03^i^	1184.85 ± 0.00^i^
S_9_	40	25	35	14.87 ± 0.02^f^	3.25 ± 0.01^e^	366.60 ± 0.03^f^	384.05 ± 0.02^f^	1406.54 ± 0.86^g^
C.V %				6.00	5.26	6.37	3.88	4.83

*Note:* Results expressed in mean ± SD. Mean do not share the same superscripts in the column are significantly different (*p* < 0.05).

Abbreviations: A, amount of barley flour (g); B, the amount of teff flour (g); C, the amount of germinated haricot bean (GHB) flour; C.V %, coefficient of variation (%); RHB, raw haricot bean (g); S_1_–S_9_, sample one to nine.

From an economic perspective, blending barley with GHB reduced production costs by 35% while maintaining over 85% of GHB's iron bioavailability. Culturally, barley‐based formulations showed greater alignment with Ethiopian dietary habits, where barley constitutes 12% of staple foods, and received higher consumer acceptance than teff‐dominant alternatives. These findings have shown barley's role as a cost‐effective, culturally appropriate carrier that preserves nutritional quality when combined with ≥ 30% GHB (Fontanini et al. 2025; Alemayehu et al. 2021).

#### 3.4.1 Iron

The present study revealed significant interactions influencing iron content in the composite flours, as demonstrated by the regression model Equation (11). The positive ternary interaction term (ABC = +14.29) indicated a synergistic effect when all three components—barley (A), teff (B), and germinated haricot bean (C)—were combined in optimal ratios, likely due to enhanced mineral bioavailability through complementary nutrient interactions. Similarly, the positive binary interaction (AC = +32.27) suggested that barley and germinated haricot bean worked together to improve iron retention, potentially by activating iron‐binding compounds during germination.

(11) $$\begin{array}{cc} \text{Iron}= & +9.98\;A+42.23B+14.49C-83.23\mathrm{AB}\\ & +32.27\;\mathrm{AC}-85\;\mathrm{BC}+14.29\;\mathrm{ABC} \end{array}$$

**FIGURE 2:**
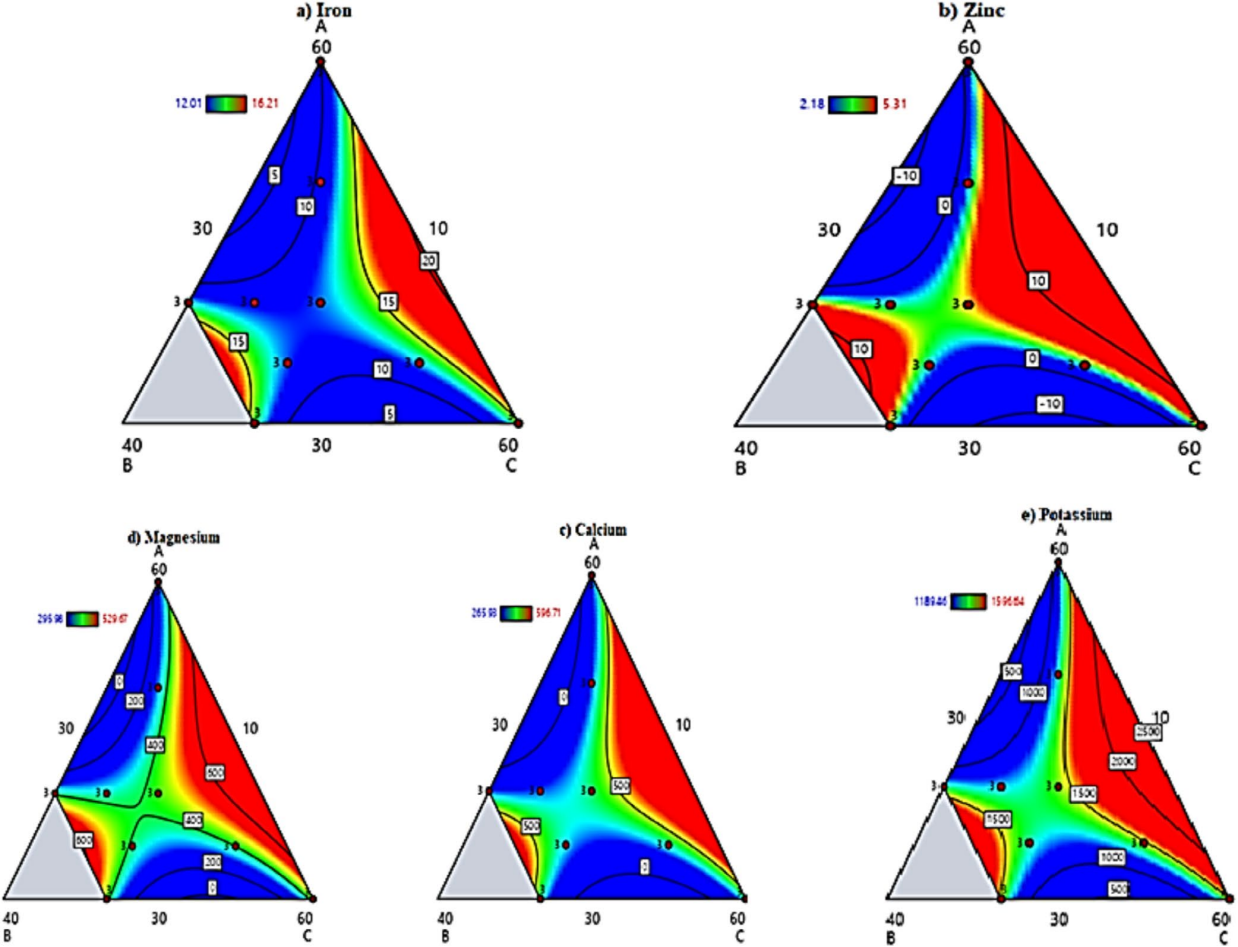
Contour plots of mineral contents of the barley‐teff‐GHB composite flours.

In contrast, strong antagonistic effects were observed in the AB (−83.23) and BC (−85.00) interactions, where combinations of barley‐teff and teff‐germinated haricot bean reduced iron bioavailability, possibly due to phytate interference or competitive mineral absorption. These findings have essential nutritional implications, particularly for pregnancy, as iron is critical in red blood cell formation, oxygen transport, and cellular functions. The formulated samples, especially S_1_ (30:10:60) and S_8_ (60:10:30) (Figure 2) with higher proportions of germinated haricot bean, showed significantly greater iron content than the control, meeting recommended daily intake levels for pregnant women (Kemal et al. 2025).

#### 3.4.2 Zinc

The zinc content in the composite flours was effectively modeled by Equation (12). Analysis of the model terms revealed important interactions between the flour components. The significant positive coefficient for teff (B = +21.50) dominated the zinc content, reflecting teff's naturally high zinc levels (5.63 ± 0.02 mg/100 g). At the same time, germinated haricot bean (C) also contributed positively (+1.78), consistent with its measured zinc content of 8.86 ± 0.08 mg/100 g. The negative coefficients for all binary interactions (AB = −0.53, AC = −0.05, BC = −0.54) indicated mild antagonistic effects when these components were combined, potentially due to competitive mineral binding or absorption interference. The negligible ternary interaction term (ABC = +0.02) suggested minimal synergistic effects when combining all three flours.

(12) $$\begin{array}{cc} \text{Zinc}\;\left(\mathrm{mg}/100\;g\right)= & +1.57A+21.50B+1.78C-0.53\mathrm{AB}\\ & -0.05\;\mathrm{AC}-0.54\;\mathrm{BC}+0.02\mathrm{ABC} \end{array}$$

Zinc plays vital roles in numerous physiological processes, including protein synthesis, cellular division, wound healing, vision, nervous system function, fetal development, and cell membrane stability. The composite flours demonstrated zinc contents ranging from 2.18 ± 0.32 to 5.31 ± 0.11 mg/100 g across different formulations, most exceeding the control sample (Figure 2). These values are nutritionally significant as they contribute meaningfully toward meeting the recommended daily zinc intake for pregnant women, which increases from 5.5 mg in the first trimester to 10 mg in the third trimester. The higher zinc levels in formulations containing greater proportions of teff and germinated haricot bean flours (which contained 5.63 ± 0.02 and 8.86 ± 0.08 mg/100 g zinc respectively, compared to barley's 3.24 ± 0.14 mg/100 g) have shown the importance of careful component selection and ratio optimization to maximize zinc bioavailability in fortified food products for maternal nutrition. These findings align with recent research by Ogunniran et al. (2024) and Abera et al. (2025).

#### 3.4.3 Calcium

The calcium content in the composite flours was effectively modeled by the Equation (13). The model revealed that teff flour (B) made by far the most significant contribution to calcium content (+879.4), consistent with its reputation as an excellent calcium source, while germinated haricot bean (C) also showed a positive though much smaller effect (+68.3). The negative coefficients for all binary interactions (AB = −22.1, AC = −2.06, BC = −21.8) indicated antagonistic effects when these components were combined, potentially due to mineral‐mineral interactions or binding with phytates. The minimal ternary interaction (ABC = +0.49) suggested negligible synergistic effects when combining all three flours.

(13) $$\begin{array}{cc} \text{Calcium}\;\left(\mathrm{mg}/100\;g\right)= & +67.\text{8A}+879.\text{4B}+68.\text{3C}-22.\text{1AB}\\ & -2.06\;\mathrm{AC}-21.8\;\mathrm{BC}+0.49\mathrm{ABC} \end{array}$$

The study found germinated haricot bean flour to have the highest calcium content among the individual components, while barley showed the lowest levels. Formulations with higher proportions of teff and germinated haricot bean flours demonstrated increased calcium content compared to control samples. The RDA level of calcium is 200 mg during the first and second trimesters, and covering 44.32%–99.45% of requirements in the third trimester, these composite flours represent a valuable dietary strategy to address calcium needs in pregnancy. The findings align with recent work by Hussain and Biswas (2024) and Manoj et al. (2025), confirming that a strategic combination of teff and germinated haricot bean flours can effectively enhance the calcium content of fortified foods for maternal nutrition.

#### 3.4.4 Magnesium

The magnesium content of the composite flours was effectively modeled by the Equation (14). Analysis of the model revealed that teff flour (B) was the dominant contributor to magnesium content (+575.9), reflecting its naturally high magnesium levels. At the same time, germinated haricot bean (C) and barley (A) showed more modest positive effects (+50.03 and + 44.7, respectively). All binary interaction terms (AB, AC, BC) exhibited negative coefficients, indicating antagonistic effects that reduced magnesium bioavailability when these flour combinations were mixed, likely due to mineral‐mineral competition or phytate interference. The minimal ternary interaction term (ABC = +0.32) suggested no meaningful synergistic enhancement when all three components were combined.

(14) $$\begin{array}{cc} \text{Magnesium}\;\left(\mathrm{mg}/100\;g\right)= & +44.\text{7A}+575.\text{9B}+50.03C-14.31\mathrm{AB}\\ & -1.43\;\mathrm{AC}-14.4\;\mathrm{BC}+0.32\mathrm{ABC} \end{array}$$

Magnesium plays vital roles in pregnancy, supporting over 300 enzymatic reactions, fetal bone development, and maternal complications like preeclampsia prevention. The composite flours demonstrated magnesium contents ranging from 295.98 ± 0.03 to 529.67 ± 0.21 mg/100 g, substantially exceeding values reported in conventional cereal products. These levels comfortably meet the pregnancy RDA of 350–400 mg/day, particularly during the critical second and third trimesters when magnesium demands peak. The formulations richest in teff and germinated haricot bean flours showed particular promise for addressing gestational magnesium needs. These findings align with recent research by Mmbando and Missanga (2024) and Thornburg and Valent (2024).

#### 3.4.5 Potassium

The potassium content of the composite flours was effectively modeled by Equation (15). This model revealed that teff flour (B) was by far the most significant contributor to potassium content (+1154.6). At the same time, germinated haricot bean (C) and barley (A) showed more modest positive effects (+57.4 and + 47.4, respectively). All binary interaction terms exhibited negative coefficients, with AB and BC showing the strongest antagonistic effects (−26.9 for both), suggesting that combining teff with either barley or germinated haricot bean reduced potassium bioavailability, possibly due to mineral‐mineral competition or binding with dietary fiber. The minimal ternary interaction (ABC = +0.56) indicated negligible synergistic effects when all three components were present.

(15) $$\begin{array}{cc} \text{Potassium}\;\left(\mathrm{mg}/100\;g\right)= & +47.\text{4A}+1154.\text{6B}+57.\text{4C}-26.\text{9AB}\\ & -0.42\;\mathrm{AC}-26.9\;\mathrm{BC}+0.56\mathrm{ABC} \end{array}$$

Potassium plays a crucial role in pregnancy by helping regulate blood pressure, maintaining fluid balance, and supporting proper muscle and nerve function. Among the raw materials, germinated haricot bean flour showed higher potassium content than barley and teff. The composite flour formulations demonstrated varying potassium levels, with formulation S_1_ (containing specific ratios of barley, teff, and germinated haricot bean) showing the highest content, while S_8_ showed the lowest (Figure 2). The developed composite flours provided approximately 33.80% of the daily potassium requirement throughout all pregnancy trimesters. This contribution aligns with the recommendations of authoritative bodies like the European Food Safety Authority (EFSA) and the World Health Organization (WHO 2022).

### 3.5 Regression Mixture Analysis for Fe, Zn, Ca, Mg, and K of Composite Flours

The ANOVA results (Table 4) have shown that mixed food products significantly influence the minerals in composite formulations. The mathematical model (special cubic) was a successful predictor of responses, with higher *F*‐values indicating variations in composite interactions.

**TABLE 4:** ANOVA values for the mineral content of composite flour samples.

Source	Fe	Zn	Ca	Mg	K
Regression	< 0.001	< 0.001	< 0.001	< 0.001	< 0.001
Linear	< 0.001	< 0.001	0.075	< 0.001	< 0.001
Quadratic	< 0.001	< 0.001	< 0.001	< 0.001	0.001
Special cubic	< 0.001	< 0.001	< 0.001	< 0.001	0.002
Lack‐of‐fit	0.218	0.342	0.156	0.121	0.087
*R* ^2^	0.994	0.929	0.995	0.989	0.983
Adj. *R* ^2^	0.974	0.941	0.961	0.927	0.959

### 3.6 Contribution of Composite Flour to the Recommended Dietary Allowance

The formulated Barley‐teff‐germinated haricot bean composite flour, derived from these composites, stands out for its high protein, iron, and calcium content compared to the recommended daily intake (RDA) for pregnant women. These composite flours provide essential nutrients such as energy, carbohydrates, and various macronutrients and micronutrient minerals crucial for maternal health.

In particular, Table 5 shows that composite flour offers 37.5% protein, 20% fat, and 12% crude fiber, meeting 100% of the calcium requirement and providing 17% of the energy pregnant women need. Moreover, consuming 100 g of the formulated flour can contribute significantly to the daily intake, offering 37.5% of the protein requirement and 5.21% of the fat requirement. This reveals the potential of composite flours as a valuable source of essential nutrients to support the dietary needs of pregnant women across different trimesters.

**TABLE 5:** Nutrient composition and percent daily nutrient contribution of prepared composite.

Nutrients (/100 g)	Obtained the results of the compositions	RDA in each trimester of pregnancy per day			Percent contribution to fulfilling the RDA by preparing composite formulations for the trimesters of pregnancy		
		First	Second	Third	First	Second	Third
Macronutrient							
Energy (kcal)	355.57–365.92	100	200–340	300–452	> 100	> 100	118.5–80.9
Carbohydrate (g)	66.57–69.58	55–135	55–175	65–175	121.0–51.8	121.0–39.7	102.4–39.7
Protein (g)	14.32–17.57	10	40	46	143.2–175.7	35.8–43.9	31.1–38.2
Fiber (g)	9.16–10.75	11	19	28	83.3–97.7	48.2–56.6	32.7–38.4
Fat (g)	2.09–3.64	ND	15	25	—	13.9–26.6	8.4–16.0
Micronutrient (mg)							
Iron	12.12–16.22	9	10–15	12–27	133.3–180	120–108	100–60
Zinc	2.07–5.13	5.5	7	10	39.6–96.6	31.1–75.9	21.8–53.1
Calcium	240.88–582.80	200	200	600	133.3–298.4	133–298.4	44.3–67.0
Magnesium	285.98–534.53	150–350	300–360	335–400	197.3–151.3	98.6–147.1	88.3–132.4
Potassium	1184.85–1599.99	3510	3510	3500	33.8–45.5	33.8–45.5	33.9–45.6.8

Abbreviations: ND, not detected in RDA; RDA, recommended dietary allowance.

### 3.7 Analysis of Anti‐Nutrients, WAC, and OAC of Composite Flours

#### 3.7.1 Tannin Content

The current study revealed tannin content through both regression modeling and experimental analysis Equation (16). The model demonstrated that teff flour (B) had the most substantial adverse effect on tannins, consistent with its low native content (0.59 mg/100 g), while barley (A) showed higher baseline levels (6.35 mg/100 g). Germination significantly reduced tannins in haricot bean flour (0.14 mg/100 g), confirming its effectiveness in anti‐nutrient mitigation.

(16) $$\begin{array}{cc} \text{Tannins}= & -2.08A-16.1B-1.8C+0.44\mathrm{AB}\\ & +0.08\mathrm{AC}+0.43\mathrm{BC}-0.01\mathrm{ABC} \end{array}$$

**FIGURE 3:**
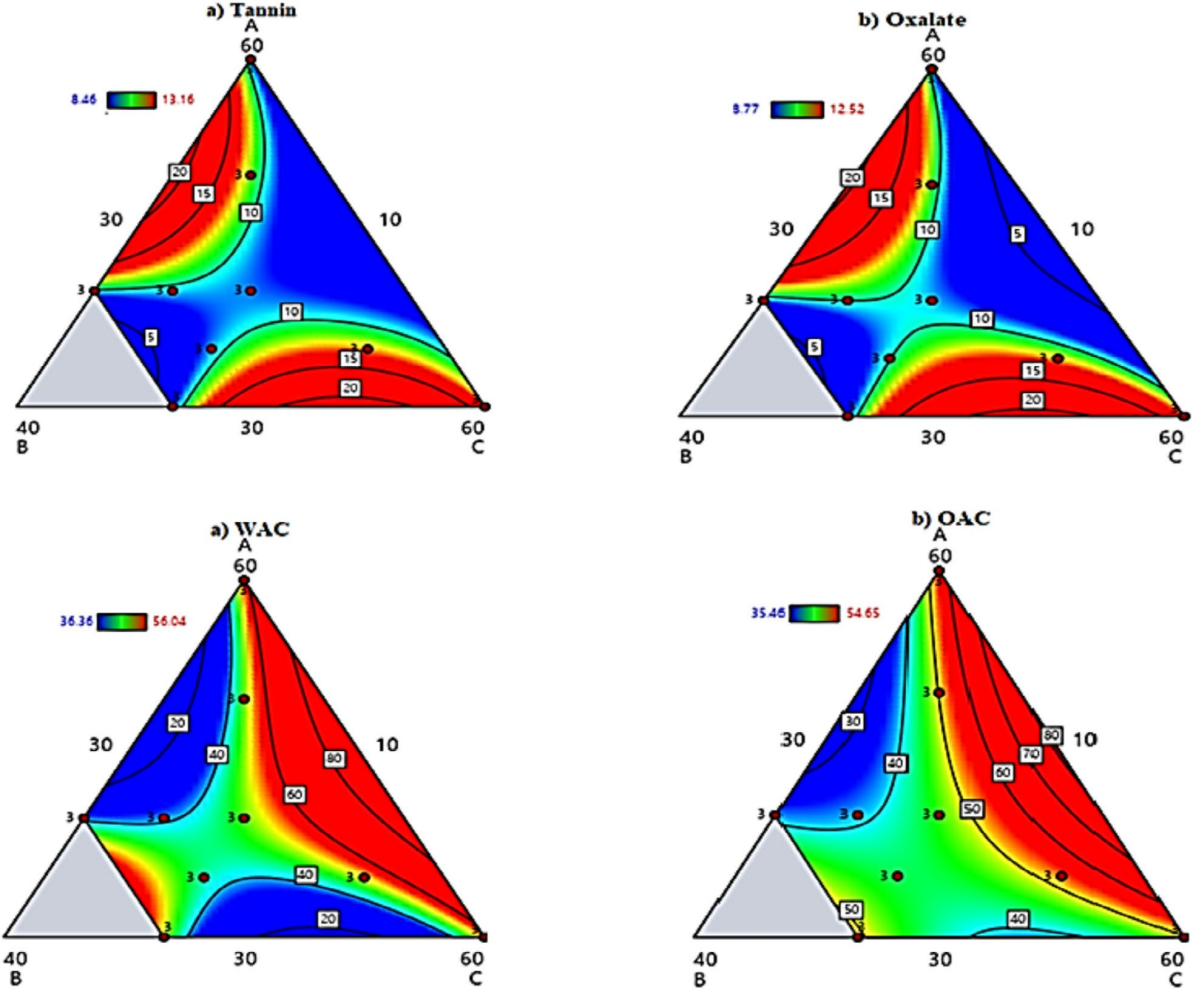
Contour plots of WAC, OAC, and overall acceptability of composite flours.

Composite formulations exhibited intermediate tannin levels (8.46–13.16 mg/100 g), with the barley‐rich S_8_ blend showing the highest content (Figure 3 and Table 6). These findings highlight two critical points: (1) barley is the primary tannin contributor in these formulations, and (2) germination processing and strategic blending with low‐tannin ingredients like teff can effectively moderate tannin levels (Fontanini et al. 2025). The results align with some literature values for barley (0.82–3.49 mg/100 g) but contrast with higher ranges reported elsewhere (9.0–70.66 mg/100 g), suggesting potential varietal or methodological differences (Mmbando and Missanga 2024).

**TABLE 6:** Results of anti‐nutrients (mg/100 g) and functional properties (%) of raw materials and composites.

Samples	Tannins	Oxalates	WAC	OAC
Raw HB	9.31 ± 0.04^a^	15.92 ± 3.20^a^	68.16 ± 0.01^b^	50.02 ± 0.55^b^
Germinated HB	0.14 ± 0.01^d^	9.56 ± 0.21^c^	80.78 ± 0.01^a^	70.48 ± 0.55^a^
Teff	0.59 ± 0.00^c^	6.20 ± 0.03^d^	50.21 ± 0.01^c^	35.03 ± 4.37^c^
Barley (C_0_)	14.35 ± 0.01^a^	12.60 ± 0.80^b^	17.27 ± 6.21^d^	15.47 ± 0.00^d^

Flour mixture ratio							
Code	A	B	C	Tannins	Oxalates	WAC	OAC
S_1_	30	10	60	8.46 ± 0.00^i^	12.52 ± 0.02^a^	56.04 ± 0.06^a^	54.65 ± 0.11^a^
S_2_	35	15	50	9.45 ± 0.00^e^	11.76 ± 0.11^b^	50.11 ± 0.01^b^	49.99 ± 0.04^c^
S_3_	40	30	30	9.68 ± 0.01^c^	9.22 ± 0.03^h^	38.29 ± 0.01^h^	38.19 ± 0.21^h^
S_4_	40	20	40	8.99 ± 0.00^g^	9.45 ± 0.01^f^	47.56 ± 1.15^d^	47.52 ± 0.15^e^
S_5_	50	15	35	12.11 ± 0.00^b^	10.67 ± 0.04^c^	40.53 ± 0.03^g^	52.09 ± 0.00^b^
S_6_	30	30	40	8.56 ± 0.01^h^	8.77 ± 0.02^i^	49.97 ± 0.05^c^	49.13 ± 0.01^d^
S_7_	35	25	40	9.09 ± 0.00^f^	9.87 ± 0.01^e^	45.81 ± 0.01^f^	45.94 ± 0.02^f^
S_8_	60	10	30	13.16 ± 0.01^a^	9.34 ± 0.10^g^	46.02 ± 0.01^e^	44.65 ± 0.10^g^
S_9_	40	25	35	9.63 ± 0.00^d^	10.06 ± 0.01^d^	36.36 ± 0.00^i^	35.46 ± 0.01^i^
C.V %				0.88	1.01	4.23	4.72

#### 3.7.2 Oxalate Content

The current study examined oxalate content in composite flours through regression modeling and experimental analysis, revealing meaningful ingredient interactions. The predictive model Equation (17) showed that all individual components—barley (A = −1.29), teff (B = −13.9), and germinated haricot bean (C = −1.11)—contributed to reducing oxalate content, with teff having the most substantial effect. However, binary combinations of barley‐teff (+0.36) and teff‐GHB (+0.35) showed mild antagonistic effects, slightly increasing oxalate levels compared to their components.

(17) $$\begin{array}{cc} \text{Oxalates}= & -1.29A-13.9B-1.11C+0.36\mathrm{AB}\\ & +0.05\mathrm{AC}+0.35\mathrm{BC}-0.00\text{8ABC} \end{array}$$

Experimental measurements confirmed these findings, showing that while barley and teff had higher native oxalate levels, the composite flours exhibited 30%–45% lower oxalate content than control barley flour, falling well below the American Dietetic Association's recommended limit of 40–50 mg/100 g. This significant reduction can be attributed to the ingredients' inherent properties and processing methods like germination. The lowered oxalate content is particularly nutritionally advantageous as it minimizes oxalate's ability to bind minerals like calcium and iron, thereby enhancing their bioavailability (Verem et al. 2021; Gebru et al. 2020; Gela 2021).

#### 3.7.3 Water Absorption Capacity

The water absorption capacity (WAC) of the composite flours was modeled by the Equation (18). The regression analysis revealed that teff flour (B = +44.9) had the strongest positive effect on WAC, followed by germinated haricot bean flour (C = +2.49) and barley flour (A = +3.23). The negative coefficients for AB (−1.09) and BC (−1.03) interactions indicated antagonistic effects when these components were combined, while the negligible ternary interaction (ABC = +0.02) suggested minimal synergistic effects in three‐component blends.

(18) $$\begin{array}{cc} \mathrm{WAC}= & +3.23A+44.\text{9B}+2.49C-1.09\mathrm{AB}\\ & -0.05\mathrm{AC}-1.03\mathrm{BC}+0.02\mathrm{ABC} \end{array}$$

Experimental results showed significant variation in WAC among the raw materials, with teff flour demonstrating superior water absorption compared to barley and germinated haricot bean flours. The enhanced WAC in germinated haricot bean flour was attributed to its higher protein content and structural modifications during germination, including polysaccharide breakdown and increased sugar availability. Among the composite formulations, S_1_ (Table 6) with higher proportions of teff and germinated haricot bean exhibited the highest WAC, while S_9_ showed the lowest values (Figure 3). These findings align with previous studies by Jeong et al. (2025) and Punzalan et al. (2025), confirming that protein‐rich, germinated ingredients significantly improve the water absorption properties of composite flours.

#### 3.7.4 Oil Absorption Capacity

Analysis of the model Equation (19) revealed that teff flour (B = +37.6) had the strongest positive effect on OAC, while germinated haricot bean flour (C = +0.09) and barley flour (A = +1.05) showed minimal individual contributions. The negative coefficients for AB (−0.87) and BC (−0.79) interactions indicated antagonistic effects when these components were combined, whereas the negligible ternary interaction (ABC = +0.02) suggested limited synergistic effects in three‐component blends. The oil absorption capacity (OAC) of the composite flours was modeled by the equation:

(19) $$\begin{array}{cc} \mathrm{OAC}= & +1.05A+37.6B+0.09C-0.87\mathrm{AB}\\ & +0.05\mathrm{AC}-0.79\mathrm{BC}+0.02\mathrm{ABC} \end{array}$$

Experimental measurements showed that the formulated blends exhibited OAC values ranging from 35.46% ± 0.01% to 54.65% ± 0.11%, with formulation S_1_ (30% barley, 10% teff, 60% GHB) demonstrating the highest capacity and S_9_ the lowest (Figure 3 and Table 6). The enhanced OAC in GHB‐containing formulations was attributed to protein denaturation during germination, which increased hydrophobic binding sites for oil molecules. The composite flours showed superior OAC (36.26%–99.75%) compared to individual components, confirming their potential to improve flavor retention, mouth feel, and nutrient density in food products (Fontanini et al. 2025).

### 3.8 Regression Mixture Analysis for Anti‐Nutrients, WAC, and OAC of Composites

The present study employed a specialized cubic regression mixture model and ANOVA analysis (Table 7) to assess composite flour samples' anti‐nutrient content and functional characteristics. The cubic model was well‐suited for evaluating anti‐nutrients and functional properties.

**TABLE 7:** ANOVA results for anti‐nutrients, functional properties of composite flours.

Source	Tannin	Oxalate	WAC	OAC	
Regression	< 0.001	< 0.001	< 0.001	< 0.001	Significant
Linear	< 0.001	< 0.001	0.075	< 0.001	
Quadratic	< 0.001	< 0.001	< 0.001	< 0.001	
Special cubic	< 0.001	< 0.001	< 0.001	< 0.001	
Lack‐of‐fit	0.218	0.342	0.156	0.121	Nonsignificant
*R* ^2^	0.994	0.929	0.995	0.989	
Adj. *R* ^2^	0.974	0.941	0.961	0.927	

Abbreviations: OAC, oil absorbance capacity; WAC, water absorption capacity.

### 3.9 Response Optimization of Variables by Overlaid Contour Plot

**FIGURE 4:**
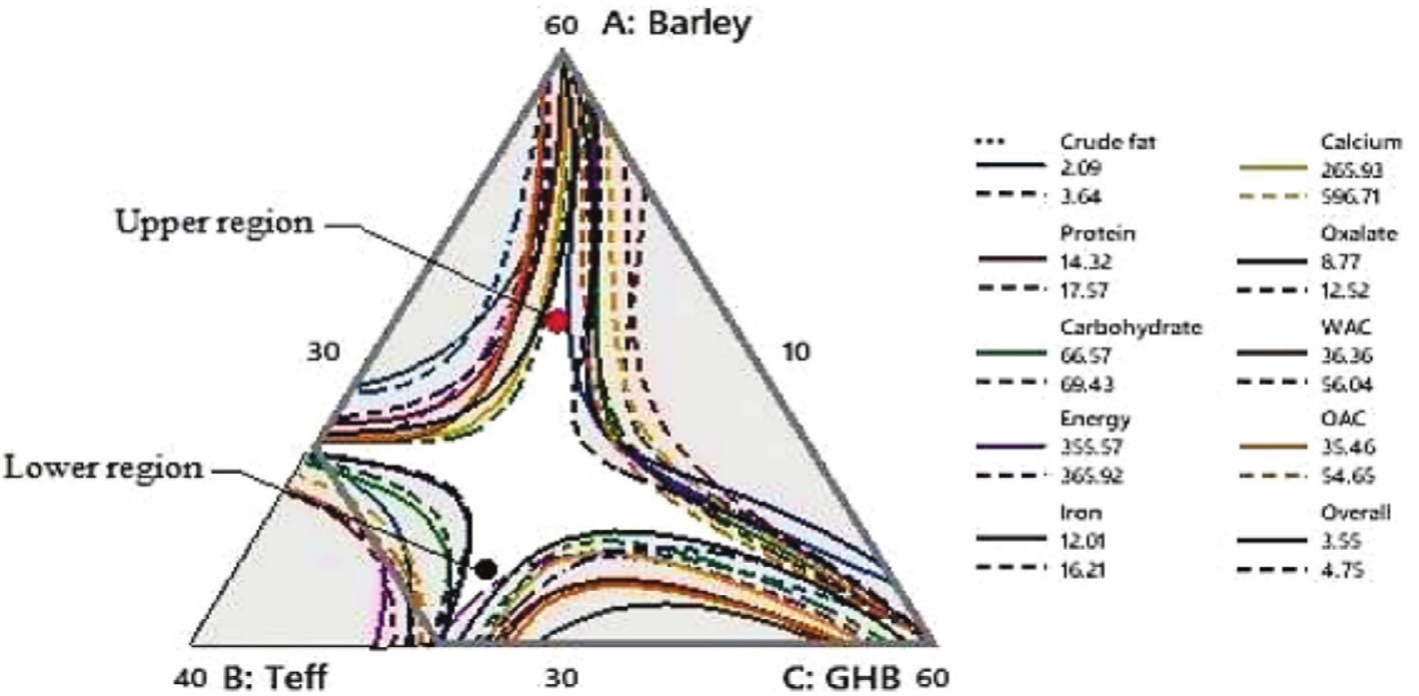
Overlaid contour plots for barley‐teff‐germinated haricot bean interactions, which contain crude fat, crude proteins, carbohydrates, energy, Fe, Ca, oxalates, WAC, OAC, and OA; the white area shows the “sweet spot” s that optimizes the response variables listed in the legend.

**FIGURE 5:**
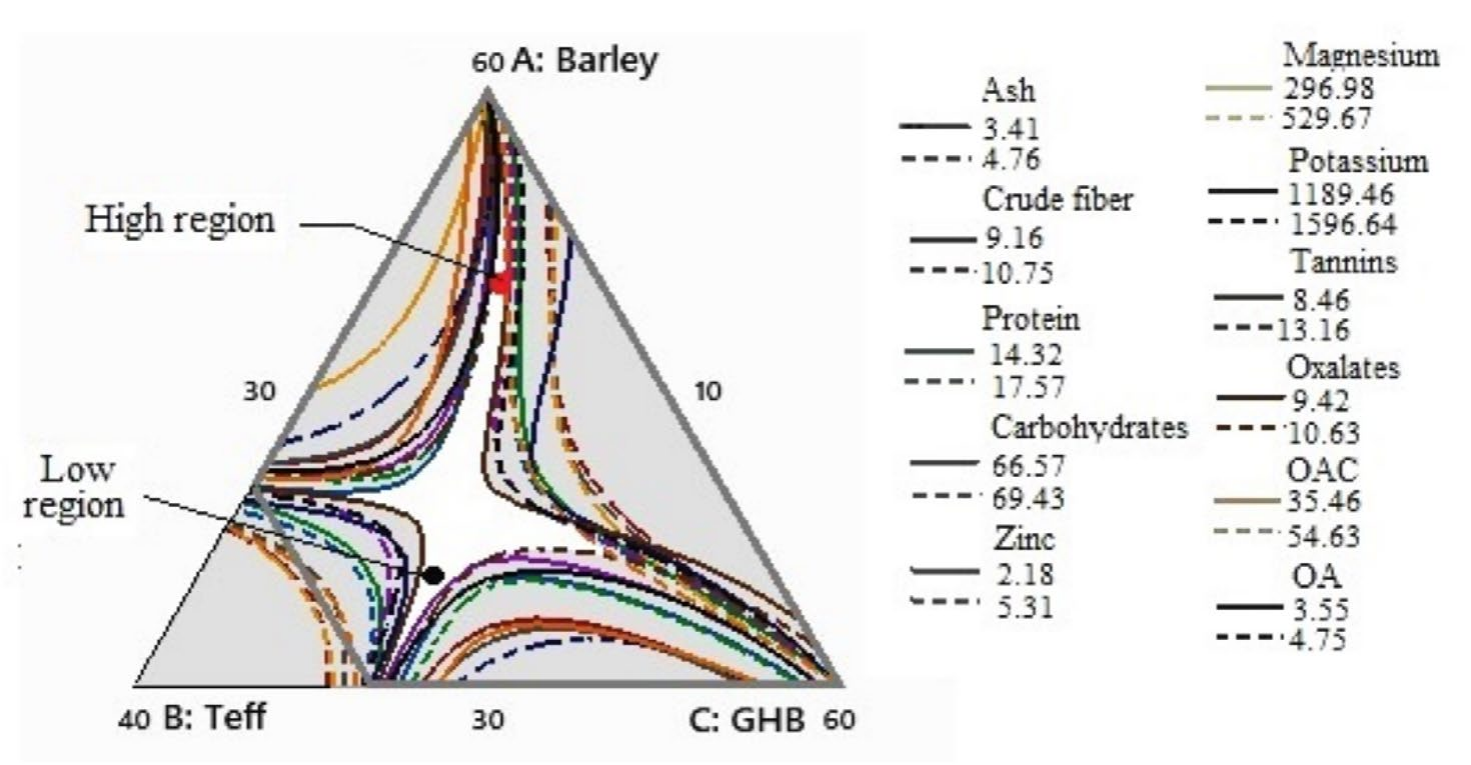
Overlaid contour plots for barley‐teff‐germinated haricot bean interactions, which contain ash, crude fiber, crude proteins, carbohydrates, Zn, Mg, K, tannins, oxalates, OAC, OA; the white area shows the “sweet spot” that optimizes the response variables listed in the legend.

This optimization aimed to identify the ideal blending ratios of barley, teff, and germinated haricot bean (GHB) that simultaneously maximize nutritional quality, minimize antinutrients, and maintain desirable functional properties for porridge formulation. Using Minitab‐generated overlaid contour plots (Figures 4 and 5), the “white sweet spot” is the overlapping region where all target responses (crude protein, minerals, WAC/OAC, and sensory acceptability) were optimized within specified constraints.

Key findings revealed two optimal composition ranges: barley (33.88–35.23 g), teff (10.75–14.45 g), and GHB (50.32–55.37 g), which balanced macronutrients (crude fat: 4.06 g/100 g; carbohydrates: 66.7 g/100 g) with iron bioavailability (14.2–14.5 mg/100 g) and reduced oxalates (3.5–7.5 mg/100 g); and (2) barley (30.4–49.44 g), teff (11.9–15.82 g), and GHB (34.74–57.67 g), which further optimized zinc (5.06–9.06 mg/100 g), potassium (1497–1772 mg/100 g), and overall acceptability (4.6–5.5/9). These “sweet spots” were selected because they represented the scientifically validated compromise between competing parameters (e.g., higher GHB increased protein but also tannins), ensuring the final blends met all criteria for nutritional, functional, and sensory quality in a cost‐effective formulation.

### 3.10 Optimization of Variables Using Response D‐Optimizer

A constrained D‐optimal mixture design was applied to optimize the proportions of barley, teff, and germinated haricot bean (GHB) flour, generating four optimal composite blends (Table 8). The D‐optimal mixture design aimed to identify composite flour ratios that simultaneously: (1) maximized protein, essential minerals (Fe, Zn, Ca, K), and functional properties (WAC, OAC); (2) minimized antinutrients (tannins, oxalates); and (3) fixed carbohydrates and moisture at targets for energy and stability. Composite desirability scores (0.90–0.97) prioritized blends meeting all criteria, with sensory acceptability ≥ 5.4/9 ensuring practical adoption.

**TABLE 8:** Optimized variables using response D‐optimizer.

Composite flour component ratios	Predicted responses and its values		Desirability
1. Components (in g)	Crude protein (g/100 g)	17.09	0.8073
Barley: 45.76	Ash (g/100 g)	5.45	1.0000
Teff: 14.77	Carbohydrate (g/100 g)	66.70	0.4662
Germinated haricot bean: 39.47	Fe (mg/100 g)	14.20	0.9985
	Zn (mg/100 g)	8.51	1.0000
	Ca (mg/100 g)	559.11	1.0000
	K (mg/100 g)	1725.44	1.0000
	Tannin (mg/100 g)	8.03	1.0000
	Oxalate (mg/100 g)	3.51	1.0000
	WAC (%)	61.84	1.0000
	OAC (%)	63.53	1.0000
	Overall acceptability	5.45	1.0000
Composite desirability			0.9149
2. Components (in g)	Crude fiber (g/100 g)	10.98	1.0000
Barley: 46.06	Crude protein (g/100 g)	17.10	0.8550
Teff: 14.82	Carbohydrates (g/100 g)	66.70	0.4671
Germinated haricot bean: 39.12	Fe (mg/100 g)	14.21	1.0000
	Zn (mg/100 g)	8.55	1.0000
	Ca (mg/100 g)	560.18	1.0000
	K (mg/100 g)	1729.19	1.0000
	Mg (g/100 g)	509.27	1.0000
	Tannin (mg/100 g)	8.04	1.0000
	Oxalate (mg/100 g)	7.50	1.0000
	WAC (%)	62.18	1.0000
	OAC (%)	63.93	1.0000
	Overall acceptability	5.45	1.0000
Composite desirability			0.9318
3. Components (in g)	Crude protein (g/100 g)	17.09	0.8519
Barley: 53.64	Carbohydrates (g/100 g)	66.70	0.4662
Teff: 11.01	Fe (mg/100 g)	14.20	0.9985
Germinated haricot bean: 57.60	Zn (mg/100 g)	9.06	1.0000
	Ca (mg/100 g)	575.63	1.0000
	K (mg/100 g)	1771.99	1.0000
	Tannin (mg/100 g)	8.03	1.0000
	WAC (%)	61.84	1.0000
	Overall acceptability	5.36	1.0000
Composite desirability			0.9023
4. Components (in g)	Moisture (g/100 g)	8.95	1.0000
Barley: 31.39	Ash (g/100 g)	4.78	0.8550
Teff: 11.01	Crude fat (g/100 g)	4.06	1.0000
Germinated haricot bean: 57.60	Crude protein (g/100 g)	17.47	1.0000
	Energy (kcal/100 g)	365.41	1.0000
	Fe (mg/100 g)	14.49	1.0000
	Zn (mg/100 g)	5.06	0.9211
	Ca (mg/100 g)	392.02	0.9323
	K (mg/100 g)	1497.90	1.0000
	Mg (g/100 g)	435.78	1.0000
	Overall acceptability	4.64	0.8691
Composite desirability			0.9711

The model evaluated nutritional, functional, and sensory parameters, with composite desirability scores ranging from 0.9023 to 0.9711, indicating high predictive reliability. The optimized blends varied in composition (barley: 31%–54%; teff: 11%–15%; GHB: 39%–58%) and achieved target outcomes, including high protein (17–17.5 g/100 g), iron (14–14.5 mg/100 g), and zinc (5–9 mg/100 g) content, alongside reduced antinutrients (tannins: 8 mg/100 g; oxalates: 3.5–7.5 mg/100 g). Functional properties critical for porridge quality—water absorption capacity (WAC: 61%–62%) and oil absorption capacity (OAC: 63%–64%)—were maximized, while sensory acceptability scores (4.6–5.5/9) ensured practical suitability. Predicted responses aligned with contour plot projections, validating the model's robustness (Table 9).

**TABLE 9:** Optimized proportions of porridge components by Minitab D‐optimizer.

Optimized composite porridge coding	Composite flour component composition (%)			
	Barley	Teff	GHB	Overall desirability
PMF_1_	45.76	14.77	39.47	0.9149
PMF_2_	46.06	14.82	39.12	0.9318
PMF_3_	53.64	11.04	35.32	0.9023
PMF_4_	31.39	11.01	57.60	0.9711

Abbreviations: GHB, germinated haricot bean; PMF_1_–PMF_4_, optimized composite flour porridges 1–4.

### 3.11 Sensory Evaluation of Porridge Developed From Composite Flours

The appearance of the porridge samples ranged from 3.32 ± 0.06 to 4.55 ± 0.12, with PMF_1_ containing 45.76 g of barley, 14.77 g of teff, and 39.47 g of germinated haricot bean achieving the highest mean score of 4.55 ± 0.12 (Table 10). In a recent study by Bonfili et al. (2025) and Aynalem and Duraisamy (2022), all porridge samples exhibited significant differences in appearance, with mean scores significantly higher than those of the control porridge.

**TABLE 10:** Results of sensory analysis for best‐optimized porridges and control.

Sample code	Food blending ratio, A + B + C (g)	Sensory attributes				
		Appearance	Aroma	Taste	Texture	Overall acceptability
PMF_1_	45.76 + 14.77 + 39.47	4.55 ± 0.12^a^	3.43 ± 0.07^d^	3.20 ± 0.04^d^	4.15 ± 0.05^c^	3.41 ± 0.08^d^
PMF_2_	46.04 + 14.82 + 39.12	4.02 ± 0.11^c^	3.58 ± 0.05^c^	3.66 ± 0.04^c^	3.46 ± 0.01^d^	4.57 ± 0.12^b^
PMF_3_	53.64 + 11.04 + 35.32	3.22 ± 0.06^d^	3.80 ± 0.15^b^	4.12 ± 0.49^b^	4.23 ± 0.10^b^	4.45 ± 0.03^c^
PMF_4_	31.39 + 11.01 + 57.60	4.49 ± 0.01^b^	4.88 ± 0.05^a^	4.96 ± 0.02^a^	4.67 ± 0.09^a^	4.82 ± 0.21^a^
Cs	100 g barley	3.00 ± 0.18^e^	2.98 ± 0.09^e^	3.10 ± 0.05^e^	3.12 ± 0.03^e^	3.20 ± 0.07^e^

*Note:* Means sharing different superscripts on column show significantly different (*p* < 0.05).

Abbreviations: A, barley flour; B, teff flour; C, germinated haricot bean flour; PMF, pregnant mother food.

The study also observed variations in aroma acceptance scores among porridge samples from different grains, including barley, teff, and germinated haricot beans. The samples with the highest acceptance scores were those composed of 45.76 g barley, 14.77 g teff, and 39.47 g germinated haricot bean, as well as those with 31.39 g barley, 11.01 g teff, and 57.60 g germinated haricot bean. Furthermore, all porridge samples demonstrated significantly higher taste acceptance than the control, with increased acceptance noted in samples with higher percentages of germinated haricot beans and chickpeas.

The study revealed that porridges with varying textures received scores ranging from 3.20 ± 0.04 to 4.96 ± 0.02, with the top‐performing formulation (PMF_4_) containing barley, teff, and germinated haricot bean. All porridges exhibited higher texture acceptance scores compared to the control sample. In assessing the sensory preferences of the porridge samples, PMF_4_ emerged as the most preferred formulation, garnering the highest overall acceptability. Furthermore, all formulated porridge samples demonstrated greater overall sensory acceptance than the 100% barley control porridge. The study concluded that PMF_4_, consisting of 31.39 g barley, 11.01 g teff, and 57.60 g germinated haricot bean, represents the optimal formulation for composite pregnant mother's food, with a higher composite desirability score of 0.9711.

## 4 Conclusions

This study demonstrates that locally grown, nutrient‐dense ingredients—specifically barley, teff, and germinated haricot beans—can effectively address the nutritional needs of pregnant women, providing over 50% of their recommended daily nutrient requirements. Germinated haricot beans contributed significantly to protein, calcium, magnesium, potassium, fiber, and iron, while teff enhanced iron and fiber content, and barley served as a rich carbohydrate source. Consequently, all these components enhance composite flours' nutritional value and sensory characteristics based on their unique nutrient profiles.

The raw materials were suitable for porridge preparation, and sensory evaluations confirmed high acceptability in taste, texture, and overall appeal. These findings demonstrate how locally‐sourced composite flours can effectively address maternal nutritional gaps in low‐resource settings. To maximize impact, future work should focus on: (1) scaling up production for community programs, (2) evaluating long‐term effects on pregnancy outcomes, and (3) refining processing methods to boost nutrient bioavailability. This research offers a practical, sustainable solution to combat malnutrition through adapted traditional foods.

## Author Contributions

**Ramesh Duraisamy:** conceptualization (equal), data curation (equal), software (equal), supervision (equal), validation (equal), visualization (equal), writing – review and editing (equal). **Abera Terefe:** conceptualization (equal), formal analysis (equal), investigation (equal), methodology (equal), visualization (equal), writing – original draft (equal). **Belay Haile Kebede:** validation (equal), visualization (equal), writing – review and editing (equal). **Betelhem Abera Mengistu:** conceptualization (equal), data curation (equal), visualization (equal), writing – review and editing (equal).

## Conflicts of Interest

The authors declare no conflicts of interest.

## Data Availability

The data that support the findings of this study are available on request from the corresponding author.
